# Risk Assessment and Source Apportionment of Metals on Atmospheric Particulate Matter in a Suburban Background Area of Gran Canaria (Spain)

**DOI:** 10.3390/ijerph20105763

**Published:** 2023-05-09

**Authors:** Yumara Martín-Cruz, Álvaro Gómez-Losada

**Affiliations:** 1 Independent Researcher, 35480 Agaete, Spain; 2 Departament of Quantitative Methods, Universidad Loyola Andalucía, Avda de las Universidades s/n, 41704 Dos Hermanas, Spain; aglosada@uloyola.es

**Keywords:** aerosols, heavy metals, health risks, source apportionment, positive matrix factorization

## Abstract

Concentration levels of 11 heavy metals were analyzed in PM${}_{10}$ and PM${}_{2.5}$ samples from a suburban area frequently affected by Saharan dust in which is located a school. The heavy metals risk assessment was carried out by the 2011 U.S. Environmental Protection Agency method, estimating the chronic and carcinogenic hazard levels both in adults and children. The highest level of chronic hazard was reached for Cr, with values of approximately 8 (PM${}_{10}$, adulthood), 2 (PM${}_{10}$, childhood) and 1.5 (PM${}_{2.5}$, adult age), significantly exceeding the limit value (equal to 1). Regarding the carcinogenic risk level, it was also high for Cr, with values between 10${}^{-3}$ and 10${}^{-1}$ for both study populations and particle size. For the rest of the studied metals, no health risk levels of concern were obtained. The positive matrix factorization method was used for the estimation of heavy metal emission sources apportionment. Non-exhaust vehicle emissions were the main source of Cr emissions under PM${}_{2.5}$, while industrial processes were the main source for PM${}_{10}$. Mineral dust and marine aerosol were common emission sources for both particles sizes—with different contributions. Vehicle emissions, construction and agricultural activities were the main emission sources for PM${}_{10}$, and fossil fuel combustion, road dust re-suspension and ammonium sulfate were for PM${}_{2.5}$. The results obtained in this study support the need to continue applying mitigation measures in suburban areas which are affected by nearby anthropogenic emissions, causing the consequent emission of materials hazardous to human health.

## 1. Introduction

Particulate matter (PM) is a harmful atmospheric pollutant with critical effects on human health, causing respiratory and cardiovascular diseases, sleep disorders [1], dermal cancer [2] and premature death. Furthermore, PM can impact the climate [3,4], interfering in the Earth’s radiation balance by scattering and absorbing solar radiation and acting as cloud condensation nuclei, affecting the biogeochemical cycles of marine organisms [5], acidifying oceans and lakes, and contributing to acid rain. Regarding the latter effect, PM can cause deterioration and blackening of materials, with associated economic costs.

PM is a complex composite of a wide variety of chemical species, such as heavy metals, which are not metabolized by the human body and accumulate in the soft tissues [6], causing serious respiratory and cardiovascular diseases, cancer and premature death [7]. Additionally, these metals can be absorbed through dermal contact and oral ingestion [8,9]. Children’s exposure to heavy metals leads to neurodevelopmental deficits, skeletal damage and adverse effects on sexual function and fertility [10].

Heavy metals can be emitted from several sources. Natural sources include mineral dust (Al, Fe, Si, Ti, Mn) and volcanic eruptions [11,12]. Among anthropogenic sources are non-exhaust (Ba, Fe, Cu, Cd, Zn) and exhaust vehicle emissions (Ca, Mg, Ni, Ba, Cu, Cd, Zn), biomass burning and incineration processes (K, Cd) [13], fossil fuel combustion (Ni, V) [14], metallurgical industries (Cr, Cu, Mn, Pb, Sn, Zn, Co) [15], mining activities (As, Cd, Cu, Ni, Pb, Zn) [16] and agricultural activities (Cu, As, Cd, Mn, Pb, Zn) [17,18]. Apportionment of these emission sources is crucial for the development and implementation of air quality policies; it is also to report the effects of mitigation measures [19].

The assessment of the risks of air pollutants on human health has been extensively studied in urban environments, being much scarcer in peripheral areas to urban centers. This study aims to contribute to fill in this gap by assessing the health risks and estimating the contribution of emission sources from 11 atmospheric heavy metals in a suburban area in the southeast of Gran Canaria island (Spain).

## 2. Materials and Methods

### 2.1. Area of Study

**Figure 1:**
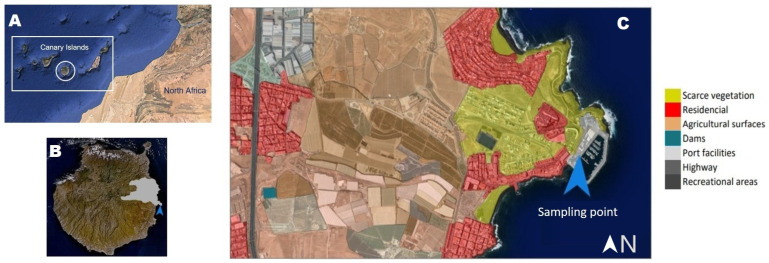
(**A**) Canary Islands. (**B**) Geographical location of the area of study (Taliarte) in Gran Canaria island. (**C**) Taliarte area.

The Canary Islands (Spain) is an archipelago located in the Atlantic Ocean which experiences intense Saharan outbreaks due to its proximity to the northeastern coast of Africa. This archipelago has eight islands, with Gran Canaria as the second most populated island. The area of study was Taliarte, an area with a suburban background in the southeast of Gran Canaria. The main sources of anthropogenic emissions in this area are vehicle (local and highway) and maritime traffic, together with agricultural activities. As for natural PM emission sources, the area of study is affected by Saharan dust and marine aerosols due to its proximity to the coast (Figure 1).

**Figure 2:**
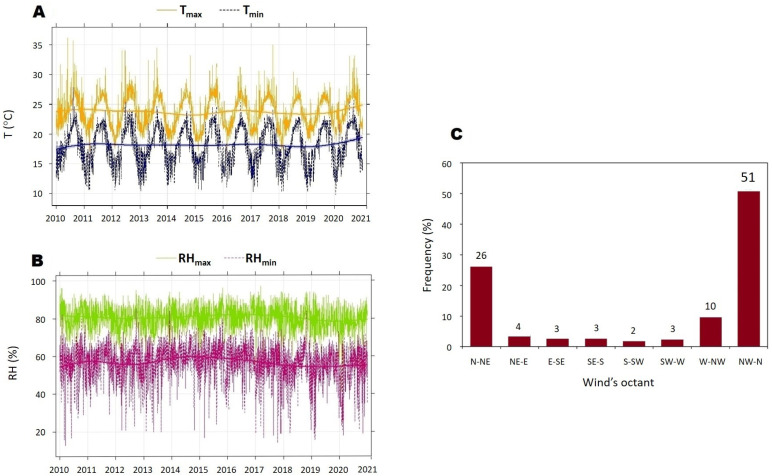
Meteorological variables of the study zone from 2010 to 2021. (**A**) Minimium and maximium temperature (T) in Canary Island. (**B**) Minimium and maximium relative humidity (RH). (**C**) Wind direction frequency by octant. Source: Aemet (Spanish Meteorology Agency).

The area of study has a mild climate, with an average annual temperature of around 21 ${}^{\circ }$C and relative humidity of approximately 69%. Figure 2 shows the temporal evolution of the temperature and relative humidity, as well as the frequency of wind direction (%) according to the wind octants between 2010 and 2021.

### 2.2. Sampling

The sampling point was at the University of Las Palmas de Gran Canaria’s Parque Científico Tecnológico building.In total, 120 samples were collected during the second semester of 2017, using high volume samplers, according to the UNE-EN 1234 normalized method. Before and after sampling, quartz filters (Whatman) were conserved at constant relative humidity (45–50%) and temperature (20–25 ${}^{\circ }$C) conditions for at least 24 h. Samples were collected on random days, except days with Saharan dust outbreaks. In these cases, the sampling was continuous from the beginning to the end of the dust event. For the forecast of these outbreaks the Barcelona Supercomputing Center (BSC-DREAM-8b and NMMB/BSC-Dust) and Skiron University numeric prediction models were consulted.

### 2.3. Sample Treatment and Chemical Analysis

A quarter of quartz sample filter was used in both PM${}_{10}$ and PM${}_{2.5}$ to analyze 11 heavy metals—Al, Ba, Cd, Cr, Cu, Fe, Mn, Ni, Ti, V and Zn. Acid digestion (2.5 mL HNO${}_{3}$:5 mL HF:2.5 mL HClO${}_{4}$) was carried out, and the samples were analyzed using a graphite furnace atomic absorption spectrometer. Two complementary analyses were performed in addition to the analysis of the eleven heavy metals in order to facilitate the subsequent identification and contribution of emission sources using positive matrix factorization (PMF): (1) Ca, Mg and K analysis (acid extraction and later analysis using a graphite furnace atomic absorption spectrometer), and (2) analysis of soluble species. To the latter, two-quarters of the sample filter were extracted with deionized water (resistivity: 18.2 MΩ-cm ) and ultrasonicated for 60 min. Ca${}^{2+}$, K${}^{+}$, Mg${}^{2+}$ and Na${}^{+}$ were analyzed with graphite furnace atomic absorption spectrometry, Cl${}^{-}$ with the argentometric method. Molecular absorption UV–Visible spectrometry (APHA 4500-NO-3B method) was used for NO${}_{3}^{-}$, and the turbidimetric method (EPA 9038) for SO${}_{4}^{2-}$. Finally, NH${}_{4}^{+}$ was analyzed using the nesslerization method (EPA 350.2).

### 2.4. Health Risk Assessment

The health risks were estimated using the method developed by the USEPA 2011 (U.S. Enviromental Protection Agency), analyzing the daily chemical intake through oral ingestion, exposure concentration through inhalation, and dermal absorption dose through dermal contact [20,21]. Then, non-carcinogenic risks (e.g., asthma, rhinitis, pulmonary fibrosis, among others) and carcinogenic risk were estimated for childhood and adulthood. For this, the hazardous index (HI) and total carcinogenic risk (TCR) for each exposure pathway were calculated. A detailed explanation of these parameters is given in Appendix A. The limit values for determining the chronic and carcinogenic risk level are shown in Table 1 [22].

### 2.5. Source Apportionment

To estimate the contribution of anthropogenic sources to the concentrations of the metals being studied both in PM${}_{10}$ and PM${}_{2.5}$, enrichment factors and positive matrix factorization were used. These are described next.

#### 2.5.1. Enrichment Factors (EFs)

Enrichment factors were calculated according to Equation (1) [23,24]:

(1) $$EF=\frac{{\left(X/R\right)}_{sample}}{{\left(X/R\right)}_{crustal}}$$

where *X* is the concentration of the metal being studied and *R* is that of the reference metal, this being the metal with the lowest anthropogenic contribution at sampling area. An EF lower than 10 indicates that the metal has a predominately natural origin, while if EF exceeds 100, the metal is emitted only by anthropogenic sources. EF values between 10 and 100 reveal a confluence of sources, with those of anthropogenic origin predominating [25].

As reference metal, Ti was chosen for crustal analysis. This metal was selected based on the study of the ratio ([Al]/[Ti])${}_{sample}$ vs. ([Al]/[Ti])${}_{crustal}$. If the first term is higher than the second term, Al is enriched, taking the other metal as reference. Standard composition of basalt from the Geochemical Database for Reference Materials (GEOREM) [26] was used to determine the (X/R)${}_{crustal}$ value.

#### 2.5.2. Positive Matrix Factorization (PMF)

PMF is a multivariate factorial analysis used to identify and quantify the sources of chemical species’ emissions at a receptor point using the fingerprint of these sources. It is the most widely used receptor-oriented model because it does not require much previous information on emission sources, enabling it to be applied to numerous studies of source apportionment [19]. This analysis, like any receptor model, is based on the principle of mass conservation between concentrations of measured species and source profiles (Equation (2)) [27,28].

(2) $$X_{ij}=\sum_{k=1}^{p}\left(g_{ik}\cdot f_{kj}\right)+e_{ij}$$

The matrix of concentrations $X_{ij}$ corresponds to a set of measured data on *i* samples of *j* chemical species, and is divided into two sub-matrices for *p* sources: the $g_{ik}$ matrix shows the contribution of *k* source at *i* sample, and the matrix $f_{jk}$ contains the concentration of *j* species for *k* source (source profiles). $e_{ij}$ is the residue corresponding to each datum value. A weighted least-squares method is used to obtain the *g* and *f* values that minimize the *Q* function (Equation (3)), including the data uncertainties ($u_{ij}$) in the input matrix. *n* and *m* indicate the number of samples and chemical species analyzed, respectively [29].

(3) $$Q=\sum_{i=1}^{n}\sum_{j=1}^{m}{\left(\frac{e_{ij}}{u_{ij}}\right)}^{2}$$

The PMF was carried out with EPA PMF 5.0 software, taking into account the concentrations of species studied and associated uncertainty around input data. Among sources of error on any measurement, the error associated with the analytical procedure is the most important [30]. The calculation procedure applied in this study to determine these uncertainties as well as the procedure performed with this software, is explained in Appendix B, Appendix C and Appendix D.

## 3. Results and Discussion

### 3.1. Descriptive Statistical Analysis

The average PM${}_{10}$ concentration during the sampling period was 64.95 ± 36.11 μg·m${}^{-3}$, exceeding the limit value permitted under Directive 2008/50/EC (50 μg·m${}^{-3}$), and reaching maximum values that quadrupled this limit. These results were the consequence of intense Saharan dust outbreaks over the islands during the sampling period, together with the presence of intense marine aerosol in the zone. In the case of PM${}_{2.5}$, the average concentration was 21.43 ± 9.71 μg·m${}^{-3}$, slightly higher than the limit established by Directive 2008/50/EC (20 μg·m${}^{-3}$), where the effect of Saharan dust was less important. As for heavy metals, the statistical summaries for both PM${}_{10}$ and PM${}_{2.5}$ at the sampling site are shown in Table 2.

**Figure 3:**
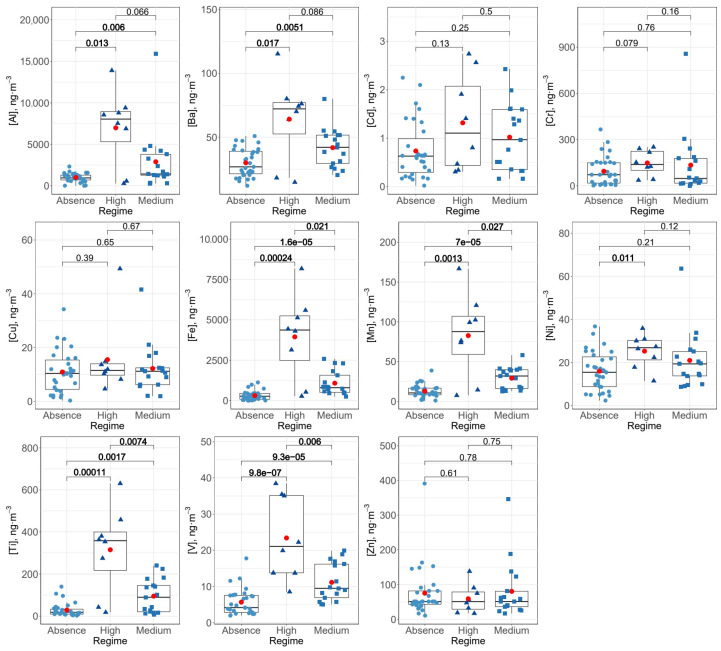
Box–whiskers plots for heavy metals studied in PM${}_{10}$ for each Saharan dust regime. Red circles indicate the average concentration and numbers show *p*-values resulting from the Wilcoxon pairwise test.

**Figure 4:**
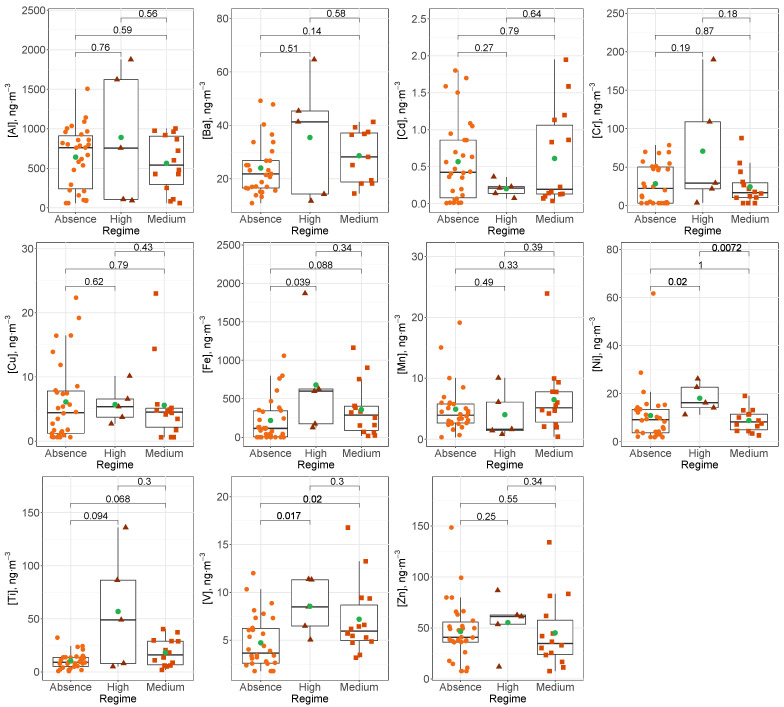
Box–whiskers plots for heavy metals studied in PM${}_{2.5}$ for each Saharan dust regimes. Green circles indicate the average concentration and numbers show *p*-values resulting from the Wilcoxon pairwise test.

As mentioned, the study area is strongly affected by Saharan dust outbreaks, impacting the concentration level of some heavy metals. This effect was observed for both crustal metals such as Al, with a coefficient of variation of 136% in contrast to 68% of variability in PM${}_{2.5}$, and anthropogenic metals such as V, with a coefficient of variation equal to 84% in PM${}_{10}$ and 58% in PM${}_{2.5}$. Figure 3 and Figure 4 show the behavior of each metal in the three Saharan dust regimes considered (absence, medium and high) for PM${}_{10}$ and PM${}_{2.5}$, respectively. The Wilcoxon pairwise test was used for the comparison of means, since a pairwise behavior of non-parametric groups was performed. A significance level of less than 0.05 was considered. The influence of Saharan dust on concentration levels of heavy metals in the fine fraction (PM${}_{2.5}$) was less significant (even in crustal metals such as Al or Mn) repeating the pattern of a greater presence of coarse particles in the dust mineral.

The predominance of fine fraction of crustal metals such as Fe and Al, during the period with non-Saharan events, with PM${}_{2.5}$ to PM${}_{10}$ ratios equal to 70% and 62.5%, respectively, could indicate their emission by anthropogenic sources such as exhaust emission from vehicle traffic [13]. Unlike these two metals, Ti and Mn showed a percentage exceeding 50% of the coarse fraction. Possible causes could be the natural local sources and non-exhaust emissions from vehicle traffic such as road dust re-suspension [31]. Two other metals linked to the latter type of metal emission are Cu and Ba, with more than 50% of their composition distributed in the coarse fraction. Both metals could have been emitted by brake pad wear, since they are typically used as filler materials in the form of barium sulfate and copper fibers [32].

**Figure 5:**
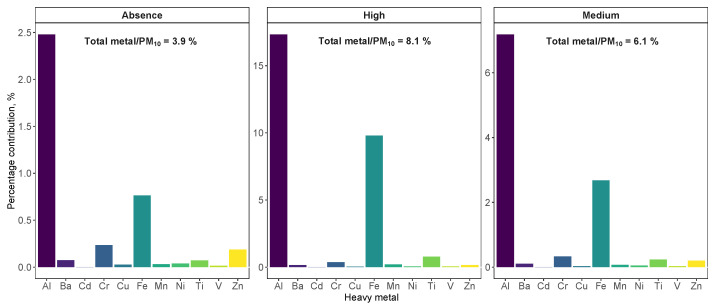
Mass percentages of each metal studied over the PM${}_{10}$ composition. The contribution of the total sum of the metals analyzed is indicated as a percentage.

**Figure 6:**
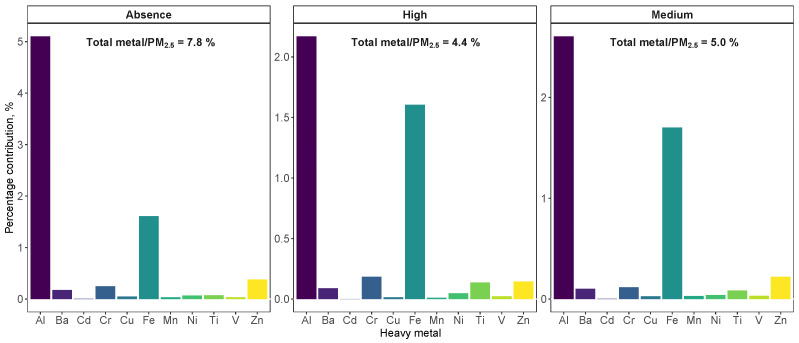
Mass percentages of each metal studied over the PM${}_{2.5}$ composition. The text indicates the contribution of the total sum of the metals analyzed.

Figure 5 shows the mass percentages of heavy metals on PM${}_{10}$ concentration. The highest contribution was obtained during intense Saharan dust episodes, due to the increase in the concentration of crustal metals such as Al, Fe and Ti. As for the anthropogenic metals such as Zn, Cr or Cd, a decrease in mass contribution was observed under these conditions as their concentration was not affected during dust outbreaks. Unlike PM${}_{10}$, the lowest percentage mass contribution for PM${}_{2.5}$ (Figure 6) was observed during intense Saharan dust outbreaks. This seems to be due to the metal composition of PM${}_{2.5}$, which was unaffected by the intensity of these Saharan dust events. Due to the mineral origin of this dust, the significant increase in metal concentration occurred in both PM${}_{10-2.5}$ fractions.

### 3.2. Health Risk Assessment

**Figure 7:**
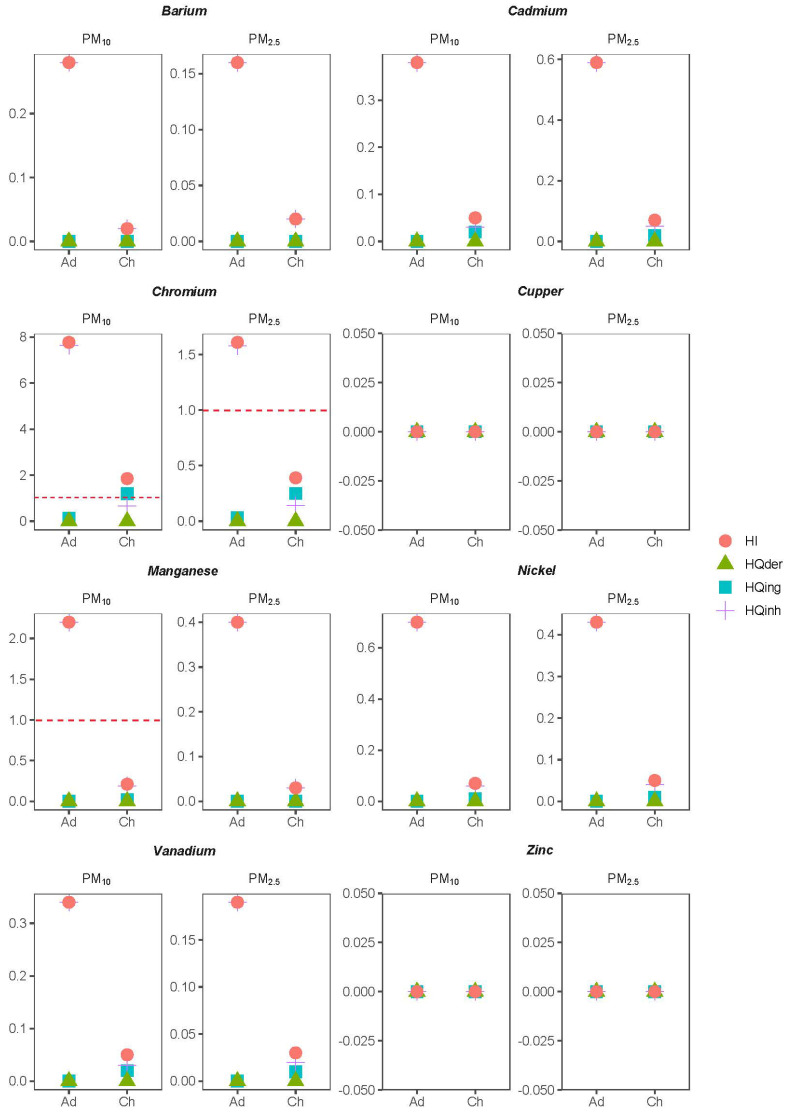
Chronic health risks at adulthood (Ad) and childhood (Ch) as measured by the values of hazardous quotient (HQ) and the hazard index by inhalation (HI${}_{inh}$), oral ingestion (HI${}_{ing}$) and dermal contact (HI${}_{der}$). A dashed red line indicates the limit value for non-carcinogenic risk.

Figure 7 shows the results obtained after the chronic risk assessment for each studied heavy metal. The main exposure pathway was inhalation, with HQ values for ingestion and dermal contact equal or very close to zero. Inhalation risk levels for Cr were the highest, significantly exceeding (up to 8 times) the USEPA limit equal to 1. The risk of causing chronic effects of Cr was reported by previous studies, such as the one conducted by [31] in a marginal area of an urban core in China. Reference [9] performed risk assessments in an urban area with high population density in China. In this study, an HQ value greater than 1 for Cr was obtained in childhood but not in adulthood. Unlike this present study, the main exposure pathway was ingestion. Likewise, in a study by [21] in the urban area of Kitahyushu (Japan), the HI value for Cr was less than 1. As for the other metals analyzed in this present research, the HQ${}_{inh}$ values were less than 1, indicating the absence of chronic risks, except for Mn (PM${}_{10}$, adulthood). In the study by [10], high values were also obtained for Mn, although at PM${}_{2.5}$.

Regarding carcinogenic risks, the main exposure pathway also was inhalation (Table 3 and Table 4). In this case, only Cd, Ni and Cr were considered, as they are classified as carcinogenic to humans (group I) by the International Agency for Research on Cancer. Previous studies [31] demonstrated the carcinogenic effect of the former metals, with TCR values higher than 10${}^{-6}$ (USEPA limit). Conversely, in this study, TCR${}_{inh}$ values for both Ni and Cd were around this limit, especially in adulthood, considered a low risk level. A similar situation was obtained in the study by [9]. In the case of Cr, high carcinogenic risk was reached both in the childhood and adulthood study in PM${}_{2.5}$ and PM${}_{10}$. These carcinogenic risks were also reported by other studies such as [8].

### 3.3. Source Apportionment

#### 3.3.1. Enrichment Factors

**Figure 8:**
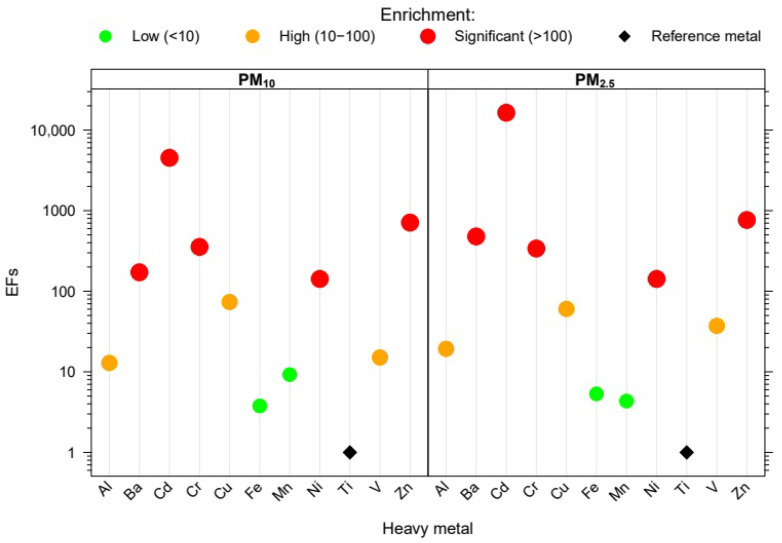
Crustal enrichment factors for the metal species studied at the sampling site. The y-axis is shown as a logarithmic scale for clarity. The enrichment scale is represented by different colors and point size. The black diamond indicates the reference metals considered.

EFs${}_{crustal}$ for metal species studied are shown in Figure 8. Cd, Cr, Cu, Ni and Zn were emitted by anthropogenic sources in PM${}_{10}$ and PM${}_{2.5}$, with values higher than 100. The EF${}_{crustal}$ for Ca was greater than 10, implying that this metal was also emitted by anthropogenic sources, such as construction activities and vehicle traffic. In the case of Na, the EF${}_{crustal}$ was remarkable, with a value higher than 100. This situation showed the influence of anthropogenic sources, such as exhaust emissions from vehicle traffic, since these high values were also observed for PM${}_{2.5}$. The case of Al and V is worth highlighting. In the PM${}_{10}$, these metals showed an EF${}_{crustal}$ very close to 10 (9.8 and 12.9, respectively), which corresponds to a predominance of crustal sources combined with anthropogenic sources. Analyzing the values achieved in PM${}_{2.5}$ for these metals, the EFs${}_{crustal}$ were higher (14.1 for Al and 37.8 for V), implying that both showed a greater contribution of anthropogenic sources in the fine fraction.

#### 3.3.2. Positive Matrix Factorization

After application of EPA PMF 5.0, six and five factors were obtained as the optimal solution for PM${}_{10}$ and PM${}_{2.5}$, respectively. The high values of the correlation factor for the scatter plots between the observed and predicted PM concentrations (0.94 for PM${}_{10}$ and 0.80 for PM${}_{2.5}$) and the lack of collinearity among factors verified the suitability of the solution adopted.

- PM${}_{10}$ emission sources

The percentage contribution of each factor to the concentration of the studied metal species is shown in Table 5. The factors are listed from highest to lowest contribution to the concentration of PM and whose sum is equal to 100.
The first factor showed high contributions of Al, Ti and Mn (>60%) and, to a lesser extent, contributions of Fe, V, Ba and K (between 20 and 40%), which have a predominantly mineral origin [33]. According to the change in the contribution of this factor, this crustal matter was dominated by the Saharan dust outbreaks, with important contribution values observed during these events. Likewise, high contributions of Cl${}^{-}$ and NO${}_{3}^{-}$, possibly due to the presence of halite and sodium nitrate (aged marine aerosol) were observed. These two ions were also explained by the second factor, which may be associated with construction activities carried out during the sampling period. Both Cl${}^{-}$ and NO${}_{3}^{-}$ are typically used as Portland cement additives to accelerate setting times, among other benefits [34]. The presence of Ba, Cu, Mn and Ni in this factor could be due to exhaust emissions from vehicles used on construction sites, used as diesel additives [35] and emitted as combustion sub-products [31,36]. Finally, Cl${}^{-}$ was also explained by the third factor, as was the Na${}^{+}$ and a slight contribution of SO${}_{4}^{2-}$, which suggests that this factor could corresponds to marine aerosol emission [30], due to the sampling site’s proximity to the coast.
The fourth factor is entirely accounted for by vehicle emissions, with a predominance of non-exhaust emissions. The intense Fe contribution in this factor can be explained by wear on brake pads, since it is their main component [32]. Other elements explained by this factor and associated with these emissions are Ba (used as filler material and considered a tracer [37]), Cu (used in reinforcement fibers [38]) and Al and Cr (employed in abrasives [38]). The second type of non-exhaust emissions corresponds to road dust re-suspension from the circulation of vehicles, with high percentages of Ca [39], K and V. The third type is due to tire wear, which could explain the contribution of this factor to the concentration of Zn, used as a vulcanization agent and the main source of ambient Zn [39]. Exhaust emissions may also be the cause of emission of Cd and Zn due to the combustion of lubricating oil [40,41] and of Mn used as a catalyst (Mn${}_{2}$O${}_{3}$) and petrol additive [42].
The fifth factor was referred as agricultural activities and traffic, and is the main source of NH${}_{4}^{+}$ emission from the use of fertilizers and manure [43]. These agricultural activities could also be responsible for high contributions of Cd and K. The proximity of the highway accounts for the contributions of metals such as Ba, Cu, Zn and Al.
The sixth factor explained a significant percentage of Cr and, to a lesser extent, Ni, which would indicate a possible industrial source [15]. Likewise, the mechanical abrasion and sanding works in the port [44] and emissions from the aircraft engines that continuously circulate in the area could also be considered a source of Cr emission.

- PM${}_{2.5}$ emission sources

The percentage contributions for each of the factors obtained in PM${}_{2.5}$ are shown in Table 6. As in the case of PM${}_{10}$, the total sum of the contributions is equal to 100. The high Ni loading in addition to V and SO${}_{4}^{2-}$ in the first factor were indicative of emissions from fossil fuel combustion [45]. The low V/Ni ratio, equal to 0.17, showed an additional source of Ni, such as emissions from motor vehicles, particularly, the diesel-powered ones. This fact was confirmed by the presence of other metals such as Ba, Cu and Mn.
The second factor may correspond to the road dust re-suspension due to high Fe and Ca presence, while the third factor was attributed to mineral dust, as it revealed high percentages of Al, Mn and Ti, which have a predominantly crustal origin, as already commented in the case of PM${}_{10}$.
The fourth factor was characterized by a high NH${}_{4}^{+}$ loading, more than 80%. As already mentioned for PM${}_{10}$, agricultural and livestock activities around of the sampling site were two significant sources of this ion. The NH${}_{4}^{+}$/SO${}_{4}^{2-}$ molar ratio was equal to 1.6, indicating that the total ammonium was neutralized by sulfates, since the molar ratio was 2:1. The slight excess of the latter anions could be due to emissions from vehicle traffic, since the presence of Ba, Ti and K was also observed. Following [46], traffic emissions are considered a major sources of these inorganic ions in urban areas, so that the factor was cataloged as “ammonium sulfate + traffic”.
Marine aerosol also contributed to the PM${}_{2.5}$ concentration, but very slightly, constituting the fifth factor and the one with the lowest percentage. This factor was characterized by high Na, Mg and K loading. It is a marine aerosol polluted by anthropogenic sources such as traffic, as indicated by the high contributions of Cd and Al.

## 4. Conclusions

In this study, PM${}_{10}$ and PM${}_{2.5}$ samples were collected in the University of Las Palmas de Gran Canaria’s Parque Científico Tecnológico building (Gran Canaria, Spain). This is a coastal and suburban area with frequent Saharan dust outbreaks. Air quality of this area was acceptable, with PM${}_{10}$ and PM${}_{2.5}$ concentration levels below Directive 2008/50/EC limits under normal conditions. Chemical characterization of the samples was carried out and the health risk assessment and sources apportionment of eleven heavy metals were performed. The highest risk levels were obtained for Cr, both for its chronic and carcinogenic effects, exceeding the established limits by USEPA. Cr emissions could come from the industrial activities states near the sampling point and from the wharf activities. Likewise, the proximity of a school to the sampling point reveals an increased exposure of children to these emissions due to outdoor activities. Based on this, the results obtained in this research support the need to implement mitigation measures in areas that are highly industrialized and seriously affected by emissions of substances that pose a health hazard to the population.

## Figures and Tables

**Table 1:** Risk level according to chronic and carcinogenic risks and values for hazardous index (HI) and total carcinogenic risk (TCR).

Type of Risk	Value	Risk Level
Chronic (non-carcinogenic)	HI ≥ 1	Causal effects
	HI < 1	Non causal effects
Carcinogenic	TCR ≥ 10${}^{-1}$	Very high
	10${}^{-3}$ ≤ TCR <10${}^{-1}$	High
	10${}^{-4}$ ≤ TCR < 10${}^{-3}$	Moderate
	10${}^{-6}$ ≤ TCR < 10${}^{-4}$	Low
	TCR < 10${}^{-6}$	Very low

**Table 2:** Average concentrations, standard deviation (μ±σ), coefficient of variation (CV) in % and maximum and minimum values (in ng·m${}^{-3}$), for each metal studied for PM${}_{10}$ and PM${}_{2.5}$.

Metal	Size	μ ± σ	CV	Max	Min
Al	PM${}_{10}$	2430.93 ± 3306.55	136	15,897.77	13.00
	PM${}_{2.5}$	645.00 ± 435.69	68	875.99	59.78
Ba	PM${}_{10}$	38.40 ± 20.35	53	115.25	12.00
	PM${}_{2.5}$	26.39 ± 12.09	46	64.63	10.86
Cd	PM${}_{10}$	0.91 ± 0.71	78	2.74	0.02
	PM${}_{2.5}$	0.54 ± 0.55	102	1.95	0.01
Cr	PM${}_{10}$	114.41 ± 136.81	120	856.66	0.58
	PM${}_{2.5}$	31.59 ± 35.36	112	189.88	3.19
Cu	PM${}_{10}$	12.01 ± 9.31	78	49.34	0.34
	PM${}_{2.5}$	5.91 ± 5.79	98	23.02	0.62
Fe	PM${}_{10}$	1062.48 ± 1617.88	152	8169.59	2.73
	PM${}_{2.5}$	304.44 ± 375.77	123	1869.68	1.58
Mn	PM${}_{10}$	27.73 ± 31.79	115	166.76	1.05
	PM${}_{2.5}$	5.26 ± 4.54	86	23.93	0.33
Ni	PM${}_{10}$	18.81 ± 10.80	57	63.53	2.35
	PM${}_{2.5}$	10.96 ± 9.78	89	61.72	1.87
Ti	PM${}_{10}$	89.85 ± 131.19	146	630.16	2.66
	PM${}_{2.5}$	17.41 ± 22.95	132	135.96	0.76
V	PM${}_{10}$	9.90 ± 8.28	84	38.45	2.16
	PM${}_{2.5}$	5.81 ± 3.37	58	16.78	1.76
Zn	PM${}_{10}$	74.81 ± 70.39	94	391.32	10.90
	PM${}_{2.5}$	46.98 ± 30.38	65	148.41	7.65

**Table 3:** TCR${}_{inh}$ values for PM${}_{10}$.

Heavy Metal	Childhood	Adulthood
Cd	9.04 × 10${}^{-7}$	3.62 × 10${}^{-6}$
Cr	5.33 × 10${}^{-3}$	2.13 × 10${}^{-2}$
Ni	9.97 × 10${}^{-7}$	3.99 × 10${}^{-6}$

**Table 4:** TCR${}_{ính}$ values for PM${}_{2.5}$.

Heavy Metal	Childhood	Adulthood
Cd	5.83 × 10${}^{-7}$	2.33 × 10${}^{-6}$
Cr	1.14 × 10${}^{-3}$	4.54 × 10${}^{-3}$
Ni	1.61 × 10${}^{-6}$	6.44 × 10${}^{-6}$

**Table 5:** Contribution of each factor obtained by the PMF model to each chemical species on PM${}_{10}$ study (in %).

Species	Factor 1	Factor 2	Factor 3	Factor 4	Factor 5	Factor 6
PM${}_{10}$	25	23	19	16	9	7
Al	72	0	4	7	16	1
Ba	24	20	0	20	30	6
Cd	0	0	11	54	27	7
Cr	0	0	20	10	0	70
Cu	0	21	19	18	26	17
Fe	30	0	0	69	0	1
Mn	65	11	2	7	7	7
Ni	6	37	0	9	13	34
Ti	85	9	0	0	2	4
V	37	16	3	30	11	2
Zn	0	0	32	19	49	0
Ca	0	35	9	37	19	0
K	20	0	2	37	33	8
Mg${}^{2+}$	15	2	68	5	8	2
Na${}^{+}$	0	0	69	14	18	0
Cl${}^{-}$	2	44	51	0	0	4
NH${}_{4}^{+}$	9	5	7	0	64	14
NO${}_{3}^{-}$	5	42	3	17	19	14
SO${}_{4}^{2-}$	0	14	13	35	39	0

**Table 6:** Contribution of each factor obtained by the PMF model to each chemical species on PM${}_{2.5}$ (in %).

Species	Factor 1	Factor 2	Factor 3	Factor 4	Factor 5
PM${}_{2.5}$	44	25	16	11	4
Al	0	5	63	1	32
Ba	34	0	32	24	10
Cd	0	52	0	2	45
Cr	7	48	10	0	35
Cu	18	53	0	18	11
Fe	0	82	10	0	8
Mn	38	0	41	8	13
Ni	70	2	32	7	0
Ti	12	4	53	31	0
V	24	36	9	21	9
Ca	9	68	32	0	0
K	10	0	33	4	52
Mg	11	2	5	4	78
Na${}^{+}$	33	0	10	0	57
NH${}_{4}^{+}$	0	0	5	85	10
NO${}_{3}^{-}$	14	0	0	41	43
SO${}_{4}^{2-}$	10	34	0	24	32

## Data Availability

Not applicable.

## Appendix A. Parameters for Health Risks Assessment

### Appendix A.1. Calculation Equations

This section shows the equations used for the calculation coefficients needs for the health risk assessment, as well as the significance of each parameter and the adopted value for each them.

(A1) $$CDI_{ing}=\frac{C_{95\%}\cdot IR\cdot EF\cdot ED\cdot CF}{BW\cdot AT}$$

(A2) $$EC_{inh}=\frac{C_{95\%}\cdot ET\cdot EF\cdot ED}{AT_{n}}$$

(A3) $$DAD_{der}=\frac{C_{95\%}\cdot SA\cdot AF\cdot ABS\cdot EF\cdot ED\cdot CF}{BW\cdot AT}$$

(A4) $$C_{95\%}=exp\left(\bar{lnX}+0.5\cdot S_{lnx}^{2}+\frac{S_{lnX}\cdot H_{0.95}}{\sqrt{n-1}}\right)$$

(A5) $$HQ_{ing}=\frac{CDI_{ing}}{RfD_{o}}$$

(A6) $$CR_{ing}=CDI_{ing}\cdot SF_{o}$$

(A7) $$HQ_{inh}=\frac{EC_{inh}}{RfC_{i}\cdot 1000}$$

(A8) $$CR_{inh}=EC_{inh}\cdot IUR$$

(A9) $$HQ_{der}=\frac{DAD_{der}}{RfD_{o}\cdot GIABS}$$

(A10) $$CR_{der}=\frac{DAD_{der}\cdot SF_{o}}{GIABS}$$

### Appendix A.2. Parameter’s Values

**Table A1:** Required parameters (name and value) for health risks assessment.

Acronym	Name	Unit	Value							
Common parameters										
EF	Exposure frequency	day·year${}^{-1}$	350							
ET	Exposure time	h·day${}^{-1}$	24							
ABS	Dermal absorption factor	-	0.001							
AT	Averaging lifetime	days	365 × 70							
	(for carcinogens)									
ATn	Average lifetime	hours	365 × 70 × 24							
	(for carcinogens)									
CF	Unit conversion factor	Kg·g${}^{-1}$	10 × 10${}^{-6}$							
Parameters dependent on life stage:			Childhood				Adulthood			
IR	Ingestion rate	mg·day${}^{-1}$	200				100			
ED	Exposure duration	year	6				24			
BW	Body weight	Kg	17				70			
AT	Averaging lifetime	days	ED × 70							
	(for non carcinogens)									
ATn	Average lifetime	hours	ED × 70 × 24							
	(for non carcinogens)									
SA	Skin surface area	cm${}^{2}$	2800				5700			
AF	Skin adherence factor	mg·cm${}^{-2}$	0.2				0.07			
Parameters dependent of heavy metals			Ba	Cd	Cr	Cu	Mn	Ni	V	Zn
RfD${}_{0}$	Oral reference doses	mg·(kg·day)${}^{-1}$	0.2	0.001	0.003	0.04	0.024	0.02	0.007	0.3
RfC${}_{i}$	Inhalation reference concentration	mg·m${}^{-3}$	0.0005	0.00001	0.00001	0.04	0.00005	0.00009	0.0001	0.3
GIABS	Gastrointestinal absorption factor	-	0.07	0.025	0.0025	1	0.04	0.04	0.026	1
Sf${}_{0}$	Oral slope factor	(mg·(kg·day)${}^{-1}$)${}^{-1}$	-	6.3	0.5	-	-	-	-	-
IUR	Inhalation unit risk	(μg·m${}^{-3}$)${}^{-1}$	-	-	0.084	-	-	-	-	-

## Appendix B. Calculation Procedure with EPA PMF 5.0

Once the input data were entered, the signal to noise (S/N) was studied for each variable, indicating if the variability of the measurements is real or within data noise [47]. This parameter can be interpreted as the relationship between concentrations of studied chemical species and uncertainties, allowing for classification of studied elements in the follow three categories. Species with S/N ≥ 2 were considered as “strong variables”, which indicates good quality data. Species with S/N between 0.2 and 2 were categorized as “weak variables”, which can indicate insufficient variations in the total concentration by them, contributing to noise on obtained results. In these cases, threefold uncertainty was employed with them by the model. Finally, species with S/N < 0.2 were labeled as “bad variables” and were excluded from analysis. In the majority of species in this study, S/N > 2 were obtained, except in the following cases: Cu and Cd were considered weak both in PM${}_{10}$ and PM${}_{2.5}$; Zn was referred to as weak in PM${}_{10}$ and bad in PM${}_{2.5}$; finally, Ti and Ni were considered as weak only in PM${}_{2.5}$. Values of S/N for each variable are shown in Table A2.

As already mentioned, the PMF model is applied to identify the number of emission sources in a specific receptor area. To determine the optimal number of factors, the following criteria were applied. Firstly, the value of Q theoretical, calculated as nm − p(n + m),was compared with the values of Q true and Q robust, which were parameters provided by the model. A good fit is obtained when these three parameters are similar to each other [48]. Secondly, scaled residuals were assessed. They are defined as the ratio of model residuals, e${}_{ij}$ and uncertainty associated by the input data. For good fit, these residuals should be symmetrically distributed within a range of −3 to +3. Thirdly, dispersion diagrams [49] between observed value and predicted value and G diagrams were evaluated. These last indicate the existence of collinearity among sources (or factors) [50]. Several proofs were carried out, considering from 5 to 8 factors and different initial additional uncertainties (0–15%).

## Appendix C. Uncertainties Calculation for PMF

In this section, the calculation procedure of required uncertainties to PMF analysis development is explained.

### Appendix C.1. Particulate Matter

Associated uncertainties to PM${}_{10}$ and PM${}_{2.5}$ were estimated with standard UNE-EN 12341:2015 using the following equation:

(A11) $$u_{T}=\sqrt{u_{fd}^{2}+\frac{u_{m}^{2}}{V^{2}}+\frac{C^{2}\cdot u_{j}^{2}}{100^{2}}}$$

$u_{fd}$: field uncertainty (0.8 μg·m${}^{-3}$ and 2.3 μg·m${}^{-3}$ for PM${}_{10}$ and PM${}_{2.5}$, respectively).

$u_{m}$: mass uncertainty. Considering the associated uncertainty to the balance and the buoyancy effect, a value of 2.5 μg·m${}^{-3}$ was considered.

$u_{fw}$: 6.3% air flow uncertainty.

C,V: particulate matter concentration (μg·m${}^{-3}$) and sampled air volume (m${}^{3}$), respectively, for PM${}_{10}$ and PM${}_{2.5}$

### Appendix C.2. Chemical Species

Associated uncertainties to studied chemical species were estimated using one of the three following equations, according to the ratio of species concentration ($X_{ij}$) to detection limit ($LD_{j}$):

(A12) $$u_{ij}\left(X_{ij}<LD_{j}\right)=X_{ij}+2/3LD_{j}$$

(A13) $$u_{ij}\left(LD_{j}<X_{ij}<3LD_{j}\right)=0.2X_{ij}+2/3LD_{j}$$

(A14) $$u_{ij}\left(X_{ij}>3LD_{j}\right)=0.1X_{ij}+2/3LD_{j}$$

0.1 and 0.2 are empirical coefficients which were obtained by [51].

## Appendix D. Additional Results from PMF Analysis

The following table shows the categorization of the studied variables according to the signal to noise ratio (S/N) for PM${}_{10}$ and PM${}_{2.5}$.

**Table A2:** S/N ratio value for PM${}_{10}$ and PM${}_{2.5}$.

Specie	Category	S/N	Category	S/N
	**PM** ${}_{10}$		**PM** ${}_{2.5}$	
PM	Strong	10.06	Strong	7.1
Al	Strong	6.8	Strong	5.3
Ba	Strong	2.8	Strong	2.0
Cd	Weak	0.9	Weak	0.5
Cr	Strong	4.1	Weak	1.7
Cu	Weak	1.5	Weak	0.6
Fe	Strong	4.4	Strong	2.3
Mn	Strong	5.5	Strong	2.4
Ni	Strong	3.2	Weak	1.9
Ti	Strong	4.2	Weak	1.9
V	Strong	6.9	Strong	6.9
Zn	Weak	0.2	Bad	0.1
Mg${}^{2+}$	Strong	5.7	—	
Na${}^{+}$	Strong	7.9	Strong	6.4
Cl${}^{-}$	Strong	8.5	—	
SO${}_{4}^{2-}$	Strong	8.8	Strong	9.0
NO${}_{3}^{-}$	Strong	8.8	Strong	9.0
NH${}_{4}^{+}$	Strong	8.3	Strong	8.9
Ca	Strong	6.6	Strong	4.4
K	Strong	8.0	Strong	5.9
Mg	—		Strong	4.5

## References

[1] Li L., Zhang W., Xie L., Jia S., Feng T., Yu H., Huang J., Qian B. (2020). Effects of atmospheric particulate matter pollution on sleep disorders and sleep duration: A cross-sectional study in the UK biobank. Sleep Med..

[2] Kim K.E., Cho D., Park H.J. (2016). Air pollution and skin diseases: Adverse effects of airborne particulate matter on various skin diseases. Life Sci..

[3] Kaufman Y.J., Tanré D., Boucher O. (2002). A satellite view of aerosols in the climate system. Nature.

[4] Zhang H., Hu J., Kleeman M., Ying Q. (2014). Source apportionment of sulphate and nitrate particulate matter in the Eastern United States and effectiveness of emission control programs. Sci. Total Environ..

[5] Li H., Chen Y., Zhou S., Wang F., Yang T., Zhu Y., Qingwie M. (2021). Change of dominant phytoplankton groups in the eutrophic coastal sea due to atmospheric deposition. Sci. Total Environ..

[6] Moryani H.T., Kong S., Du J., Bao J. (2020). Health risk assessment of heavy metals accumulated on PM_2.5_ fractioned road dust from two cities of Pakistan. Int. J. Environ. Res. Public Health.

[7] Kastury F., Smith E., Juhasz A.L. (2017). A critical review of approaches and limitations of inhalation bioavailability and bioaccessibility of metal(loid)s from ambient particulate matter or dust. Sci. Total Environ..

[8] Aminiyan M.M., Baalousha M., Aminiyan F.M. (2018). Evolution of human health risk based on EPA modeling for adults and children and pollution level of potentially toxic metals in Rafsanhan road dust: A case study in a semi-arid region, Iran. Environ. Sci. Pollut. Res..

[9] Liu P., Ren H., Xu H., Lei Y., Shen Z. (2018). Assessment of heavy metals characteristics and health risks associated with PM_2.5_ en Xi’an, the largest city in northwestern, China. Air Qual. Atmos. Health.

[10] Zhang H., Zhenxing M., Huang K., Wang X., Cheng L., Zeng L., Zhou Y., Jing T. (2019). Multiple exposure pathways and health risk assessment of heavy metal(loid)s for children living in fourth-tier cities in Hubei Province. Environ. Int..

[11] Morillas H., Maguregui M., Paris C., Bellot-Gurlet L., Colomban P., Madariaga J.M. (2015). The role of marine aerosol in the formation on double sulfate/nitrate salts in plasters. Microchem. J..

[12] Rodriguez S., Calzolai G., Chiari M., Nava S., García M.I., López-Solano J., Marrero C., López-Darias J., Cuevas E., Alonso-Pérez S. (2020). Rapid changes of dust geochemistry in the Saharan Air Layer linked to sources and meteorology. Atmos. Environ..

[13] Saggu G.S., Mittal S.K. (2020). Source apportionment of PM_10_ by positive matrix factorization model at a source region of biomass burning. J. Environ. Manag..

[14] Manousakas M., Diapouli E., Papaefthymiou H., Migliori A., Karydas A.G., Padilla-Alvarez R., Bogovac M., Kaiser R.B., Jaksic M., Bogdanovic-Radovic I. (2015). Source apportionment by PMF on elemental concentrations obtained by PIXE analysis of PM_10_ samples collected at the vicinity of lignite power plants and mines in Megalopolis, Greece. Nucl. Instrum. Methods Phys. Res. Sect. Beam Interact. Mater. Atoms.

[15] Sadeghi B., Choi Y., Yoon S., Flynn J., Kotsakis A., Lee S. (2020). The characterization of fine particulate matter downwind of Houston: Using integrated factor analysis to identify anthropogenic and natural sources. Environ. Pollut..

[16] Blondet I., Schreck E., Viers J., Casas S., Jubany I., Bahí N., Zouiten C., Dufréchou G., Freydier R., Galy-Lacaux C. (2019). Atmospheric dust characterization in the mining distric of Cartagena-La Unión, Spain: Air quality and health risks assessment. Sci. Total Environ..

[17] Kumar A., Chauchan A., Arora S., Tripathi A., Mohammed S., Alghanem S., Khan K.A., Ghramh H.A., Özdemir A., Ansari M.J. (2020). Chemical analysis of trace metal contamination in the air of industrial area of Gajraula (U.P.), India. J. King Saud Univ.-Sci..

[18] Tyagi V., Gurjar B., Joshi N., Kumar P. (2012). PM_10_ and heavy metals in suburban and rural atmospheric environments of Norhthern India. Pract. Period. Hazard. Toxic Radioact. Waste Manag..

[19] Sun X., Wang H., Guo Z., Lu P., Song F., Liu L., Liu J., Rose N.L., Wang F. (2020). Positive matrix factorization on source apportionment for typical pollutants in different environmental media: A review. Environ. Sci. Process. Impacts.

[20] MohseniBandpi A., Eslami A., Ghaderpoori M., Shahsavani A., Jeihooni A.K., Ghaderpoury A., Alinejad A. (2018). Health risk assessment of heavy metals on PM_2.5_ in Tehran air, Iran. Data Brief.

[21] Zhang X., Eto Y., Aikawa M. (2021). Risk assessment and management of PM_2.5_-bound heavy metals in the urban area of Kitakyushu, Japan. Sci. Total Environ..

[22] Chen R., Jia B., Tian Y., Feng Y. (2021). Source-specific health risk assessment of PM_2.5_-bound heavy metals based on high time-resolved measurement in a chinese megacity: Insights into seasonal and diurnal variations. Ecotoxicol. Environ. Saf..

[23] Shelley R.U., Morton P.L., Langing W.M. (2015). Elemental ratios and enrichment factors in aerosols from the US-GEOTRACES North Atlantic transects. Deep-Sea Res. II.

[24] Buck C.S., Aguilar-Islas A., Marsay C., Kadko D., Landing W.M. (2019). Trace element concentrations, elemental ratios and enrichment factors observed in aerosol samples collected during the US GEOTRACES eastern Pacific Ocean transect (GP16). Chem. Geol..

[25] Heldari-Farsani M., Shirmardi M., Goudarzi G., Alavi N., Ahmadi-Ankali K., Zallaghi E., Naimabadi A., Hashemzadeh B. (2014). The evaluation of heavy metals concentration related to PM_10_ in ambient air of Ahvaz City, Irane. J. Adv. Environ. Health Res..

[26] Jochum K.P., Nohl U., Herwing K., Lammel E., Stoll B., Hofmann A.W. (2007). GeOReM: A new geochemical database for reference materials and isotopic standards. Geostand. Geonalytical Res..

[27] Hopke P.K. (2016). Review of receptor modeling methods for source apportionment. J. Air Waste Manag. Assoc..

[28] Mircea M., Calori G., Pirovano G., Bellis C. (2008). European guide on air pollution source apportionment for particulate matter with source oriented models and their combined use with receptor models. EUR 30082 EN Publ. Off. Eur. Union Luxemb..

[29] Bellis C.A., Larsen B.R., Amato F., El Haddad I., Favez O., Harrison R.M., Hopke P.K., Nava S., Paatero P., Prévôt A. (2013). European Guide on Air Pollution Source Apportionment with Receptor Models.

[30] Millan-Martinez M., Sánchez-Rodas D., Sánchez de la Campa A., de la Rosa J. (2021). Contribution of anthropogenic and natural sources in PM_10_ during North African dust events in Southern Europe. Environ. Pollut..

[31] Wang J.M., Jeong C.H., Healy R.M., Sofowote U., Debosz J., Su Y., Munoz A., Evans G.J. (2021). Quantifying metal emission from vehicle traffic using real world emissions factors. Environ. Pollut..

[32] Cai R., Zhang J., Nie X., Tjong J., Matthews D.T.A. (2020). Wear mechanism evolution on brake discs for reduced wear and particulate emissions. Wear.

[33] Lokorai K., Ali-Khodja H., Khardi S., Bencharif-Madani F., Naidja L., Bouziane M. (2021). Influence of mineral dust on the concentration and composition of PM_10_ in the city of Constantine. Aeolian Res..

[34] Dorn T., Blask O., Stephan D. (2022). Acceleration of cement hydration—A review of the working mechanisms, effects on setting time, and compressive strength development of accelerating admixtures. Contruction Build. Mater..

[35] Hosseinzadeh-Bandbafha H., Tabatebaei M., Aghbashlo M., Khanali M., Demirbas A. (2018). A comprehensive review on the environmental impacts of diesel/biodiesel additives. Energy Convers. Manag..

[36] Tian H.Z., Lu L., Cheng K., Hao J.M., Zhao D., Wang Y., Jia W.X., Qiu P.P. (2012). Anthropogenic atmospheric nickel emissions and its distribution characteristics in China. Sci. Total Environ..

[37] Beddows D.C.S., Dall’Osto M., Olatunbosum O.A., Harrison R.M. (2016). Detection of brake wear aerosols by aerosol time-of-flight mass spectrometry. Atmos. Environ..

[38] Thorpe A., Harrison R.M. (2008). Sources and properties of non-exhaust particulate matter from road traffic: A review. Sci. Total Environ..

[39] Amato F., Alastuey A., Karanasiou A., Lucarelli F., Nava S., Calzolai G., Severi M., Becagli M., Gianelle V.L., Colombi C. (2016). AIRUSE-LIFE+: A harmonized PM speciation and source apportionment in five southern European cities. Atmos. Chem. Phys..

[40] Hao Y., Meng X., Yu X., Lei M., Li W., Shi F., Yang W., Zhang S., Xie S. (2018). Characteristics of trace elements in PM_2.5_ and PM_10_ of Chifeng, northeast China: Insights into spatiotemporal variations and sources. Atmos. Res..

[41] Li W., Dryfhout-Clark H., Hung H. (2020). PM_10_-bound trace elements in the Great Lakes Basin (1988–2017) indicates effectiveness of regulatory actions, variations in sources and reduction in human health risks. Environ. Int..

[42] Ghosh S., Rabba R., Chowdhury M., Padhy P.K. (2018). Source and chemical species characterization of PM_10_ and human health risk assessment of semi-urban, urban and industrial areas of West Bengal, India. Chemosphere.

[43] Kabelitz T., Ammon C., Funk R., Münch S., Biniasch O., Nübel U., Thiel N., Rösler U., Siller P., Amon B. (2020). Functional relationship of particulate matter (PM) emissions, animal species, and moisture content during manure application. Environ. Int..

[44] Wu S.H., Cai M.J., Xu C., Zhang N., Zhou J.B., Yan J.P., Schwab J.J., Yuan C.S. (2020). Chemical nature of PM_2.5_ and PM_10_ in the coastal urban Xiamen, China: Insights into the impacts of shipping emissions and health risk. Atmos. Environ..

[45] Moreno T., Querol X., Alastuey A., de la Rosa J., Sánchez de la Campa A.M., Minguillón M., Pandolfi M., González-Castanedo Y., Monfort E., Gibbons W. (2010). Variations in Vanadium, nickel and lanthnoid element concentrations in urban air. Sci. Total Environ..

[46] Xing W., Yang L., Zhang H., Zhang X., Wang Y., Bai P., Zhang L., Hayakawa K., Nagao S., Tang N. (2021). Variations in traffic-related water soluble inorganic ions in PM_2.5_ in Kanazawa, Japan, after the implementation of a new vehicle emissions regulation. Atmos. Pollut. Res..

[47] Paatero P., Hopke P.K. (2003). Discarding or downweighting high-noise variables in factor analytic models. Anal. Chim. Acta.

[48] Paatero P., Hopke P.K. (2009). Rotational tools for factor analytic models. Chemometrics.

[49] Alvi M.U., Kistler M., Mahmud T., Shahid I., Alam K., Chisthie F., Hussain R., Kasper-Giebl A. (2019). The composition and sources of water soluble ions in PM_10_ at an urban site in the Indo-Ganfetic Plain. J. Atmos. Sol.-Terr. Phys..

[50] Paatero P., Hopke P.K., Begum B.A., Biswas S.K. (2005). A graphical diagnostic method for assessing the rotation in factor analytical models of atmospheric pollution. Atmos. Environ..

[51] Zabalza J., Ogulei D., Hopke P., Lee J., Hwang I., Querol X., Alastuey A., Santamaria J. (2006). Concentration and sources of PM_10_ and its constituents in Alsasua, Spain. Water Air Soil Pollut..

